# The practicality of different eGFR equations in centenarians and near-centenarians: which equation should we choose?

**DOI:** 10.7717/peerj.8636

**Published:** 2020-02-21

**Authors:** Qiuxia Han, Dong Zhang, Yali Zhao, Liang Liu, Jing Li, Fu Zhang, Fuxin Luan, Jiayu Duan, Zhangsuo Liu, Guangyan Cai, Xiangmei Chen, Hanyu Zhu

**Affiliations:** 1 Chinese PLA General Hospital, Chinese PLA Institute of Nephrology, State Key Laboratory of Kidney Diseases, National Clinical Research Center for Kidney Diseases, Beijing Key Laboratory of Kidney Diseases, Beijing, China; 2 School of Medicine, Nankai University, Tianjin, China; 3 The First Affiliated Hospital of Zhengzhou University, Research Institute of Nephrology of Zhengzhou University, Key Laboratory of Precision Diagnosis and Treatment for Chronic Kidney Disease in Henan Province, Zhengzhou, China; 4 Central Laboratory, Hainan Branch of Chinese PLA General Hospital, Sanya, China; 5 Management Department, Hainan Branch of Chinese PLA General Hospital, Sanya, China

**Keywords:** eGFR, Centenarians, CKD-EPI equation, BIS1 equation, MDRD equation

## Abstract

**Background:**

No studies have examined the practicality of the Modification of Diet in Renal Disease (MDRD), Chronic Kidney Disease Epidemiological Collaboration (CKD-EPI) and Berlin Initiative Study 1 (BIS1) equations for the estimated glomerular filtration rate (eGFR) in a large sample of centenarians. We aim to investigate the differences among the equations and suggest the most suitable equation for centenarians and near-centenarians.

**Methods:**

A total of 966 centenarians and 787 near-centenarians were enrolled, and the eGFR was calculated using the three equations mentioned above. Agreement among the equations was investigated with the *κ* statistic and Bland–Altman plots. Sources of discrepancy were investigated using a partial correlation analysis.

**Results:**

The three equations for assessing eGFR are not considered interchangeable in centenarians and near-centenarians. Δ(MDRD, CKD-EPI) and Δ(MDRD, BIS1) increased with age, but Δ(CKD-EPI, BIS1) was relatively stable with age. Δ(MDRD, CKD-EPI) and Δ(MDRD, BIS1) were considerable in subjects with Scr levels less than 0.7 mg/dL and decreased with the Scr level. A considerable difference between CKD-EPI and BIS1 was observed for participants with Scr levels ranging from 0.5 to 1.5 mg/dL. This difference increased with Scr levels ranging from 0.5 to 0.7 mg/dL, was relatively stable for Scr levels ranging from 0.7 to 0.9 mg/dL, and decreased with Scr levels ranging from 0.9 to 1.5 mg/dL. The differences in the three comparisons were all greater in women than in men (*p* < 0.05).

**Conclusions:**

We tend to suggest the MDRD equation to calculate the glomerular filtration rate (GFR) in elderly individuals >95 years old who have no risk factors for cardiovascular disease; the BIS1 equation to calculate the eGFR for elderly individuals younger than 94 years old who have risk factors for cardiovascular disease; the CKD-EPI equation to calculate the eGFR of elderly individuals with Scr levels greater than 1.5 mg/dL; and the BIS1 equation to calculate the eGFR of older women with Scr levels less than 0.7 mg/dL.

## Introduction

The population of individuals of an advanced age, including near-centenarians and centenarians, has become the fastest-growing portion of the population worldwide, particularly in China (Zeng et al., 2017). This growth has led to considerable social and economic burdens from various diseases, such as chronic kidney disease (CKD), in this age group (He et al., 2018). Therefore, the prevention and treatment of CKD are particularly important and urgent in elderly patients. The glomerular filtration rate (GFR) is a sensitive index for evaluating renal function to aid in the evaluation of drug doses. In addition, it is an important basis for diagnosing and staging CKD, as well as for monitoring treatment (Levey & Inker, 2016). The gold standard for estimating GFR is urinary inulin clearance, which is a complicated and time-consuming process (Al-Wakeel, 2016). Several serum creatinine (Scr)-based equations for estimating the GFR have been developed and tested for many years to solve this problem. The most common equations used to assess the estimated GFR (eGFR) are the Modification of Diet in Renal Disease (MDRD), Chronic Kidney Disease Epidemiology Collaboration (CKD-EPI) and Berlin Initiative Study 1 (BIS1) equations (Levey et al., 2006; Levey et al., 2009; Schaeffner et al., 2012). However, all these equations for eGFR were not initially developed using a large sample of centenarians and near-centenarians. Thus, the practicality of the GFR prediction equations in them with CKD is questionable (Ungar et al., 2015). According to some studies, the use of the two “classical” equations (MDRD and CKD-EPI) overestimates the GFR in patients older than 70 years of age with CKD stage 3 (GFR 30–59 mL/min) compared to the isotopic method 99mTc-DTPA (Maioli et al., 2014). The BIS1 equation was developed and validated for use in older people (Schaeffner, Martus & Ebert, 2014). This equation was suggested for estimating the GFR in older patients with mild to moderate CKD and was considered more accurate than the CKD-EPI equation (Oscanoa et al., 2018). However, some studies have questioned the utility of this equation. Indeed, the BIS1 equation did not outperform the CKD-EPI equation in predicting GFR measured by iohexol clearance as the reference standard in older people (Fan et al., 2015). Additionally, some studies have shown that the CKD-EPI equation has a lower bias in predicting inulin clearance than the BIS1 equation in older patients with severe CKD (Koppe et al., 2013). Thus, an ideal equation for calculating the GFR of older people has not yet been identified.

Notably, the selection of older adults for diagnostic and therapeutic interventions related to CKD stage changes depends on which equation is used (Changjie et al., 2017). Thus, considering the discordance among these equations with regard to their respective accuracies for predicting measured GFR has practical clinical implications (Corsonello et al., 2017). Centenarians represent a unique population in experimental research because they develop all the signs and characteristics of the extreme aging process (Puca et al., 2017). However, these equations for eGFR have not yet been validated in centenarians who have extreme longevity. The aim of the present study is to compare the MDRD, CKD-EPI and BIS1 equations, assess the sources and clinical significance of discrepancies among them and suggest the most appropriate equation for specific groups of centenarians and near-centenarians.

## Materials & Methods

### Data source

We used data from the China Hainan Centenarian Cohort Study (CHCCS), which was designed to investigate centenarians’ physical and mental health statuses as well as their social condition. The researchers obtained ethical approval for the study protocol from the Ethics Committee of the Hainan branch of the Chinese People’s Liberation Army General Hospital (Sanya, Hainan) (Approval No.: 301HNLL-2016-01). Participants received an extensive description of the study and signed an informed consent form that included permission to analyze biological specimens that were collected and stored. For participants who were unable to provide complete consent because of cognitive or physical problems, surrogate consent was obtained from a close relative.

A total of 1,807 partipants were enrolled in the CHCCS, and basic information and blood biochemical indexes were available for 1753 of these participants. Details of the participant recruitment process and study scheme are shown in Table 1. CKD staging was performed according to the Kidney Disease: Improving Global Outcomes (KDIGO) clinical practice guidelines: eGFR ⩾ 90, stage 1; 60 ⩽ eGFR < 90, stage 2; 45 ⩽ eGFR < 60, stage 3a; 30 ⩽ eGFR < 45, stage 3b; 15 ⩽ eGFR < 30, stage 4; and eGFR < 15, stage 5 (units: mL/min/1.73 m^2^).

**Table 1:** Participant recruitment in CHCCS.

	Centenarians	Near-centenarians	Total
Age inclusion criteria	Over 100 years old	80–100 years old	–
Recruited	1,004	803	1,807
Men: *N* (%)	176 (17.53%)	326 (40.60%)	502 (27.78%)
Women: *N* (%)	828 (82.47%)	477 (59.40%)	1,305 (72.22%)
Both basic information and blood biochemical index are available	966	787	1,753
Men: *N* (%)	175 (18.12%)	316 (40.15%)	491 (28.00%)
Women: *N* (%)	791 (81.88%)	471 (59.85%)	1,262 (72.00%)

**Notes.**

Abbreviations CHCCS the China Hainan Centenarian Cohort Study

Although no immediate plan exists to make the data freely available in the public domain, specific proposals for further collaboration are welcome. For further information, please contact the corresponding authors via e-mail (hanyuzhu301@126.com).

### Laboratory assays

Blood samples were obtained after the patients had fasted for 12 h and rested for at least 15 min and were stored separately in refrigerated containers until they were analyzed on the same day. Complete blood cell counts were measured with Sysmex XT4000i (Sysmex Corporation, Kobe, Japan). Blood biochemical parameters were measured with a fully automatic biochemical autoanalyzer (Cobas 8000; Roche Products Ltd. Basel, Switzerland). The Roche assay of creatinine is standardized by and traceable to IDMS methods.

### Analytical approach

First, we analyzed the demographic and clinical characteristics of the participants and calculated the prevalence of the selected disease. Normally distributed data were presented as the means ± standard deviation and were compared using unpaired Student’s *t*-tests. Nonnormally distributed data were presented as medians with corresponding 25th and 75th percentiles (interquartile ranges) and compared using Mann–Whitney U tests. We performed partial correlation analyses to evaluate correlations between equations related factors and the differences of these three equations and to identify how they influenced the discordance among them. *P* values less than 0.05 were considered statistically significant. We used a graphic approach by plotting scatter diagrams to evaluate the relationship between Scr levels and differences in the values obtained from these three equations, and we used locally weighted scatterplot smoothing nonparametric regression methods to investigate the impact of age on the differences in the values obtained from these three equations. Because the categories were ordered, agreement among the three different equations was quantified using the linear weighted *κ* statistic. The criteria used to evaluate agreement were as follows: 0 ⩽ *κ* value < 0.21, slight agreement; 0.21 ⩽ *κ* value < 0.41, fair agreement; 0.41 ⩽ *κ* value < 0.61, moderate agreement; 0.61 ⩽ *κ* value < 0.81, substantial agreement; and 0.81 ⩽ *κ* value ⩽ 1.00, almost perfect agreement (Ludbrook, 2010). Unpaired Student’s *t*-tests were used to investigate the impact of sex on differences (Δ) among these three equations.

Statistical analyses were performed using SPSS software (version 19.0 SPSS, Chicago IL, USA), GraphPad Prism software (Vision 6, San Diego, CA, USA) and Medcalc for Windows (version 9.3.9.0 Medcalc software, Mariakerke, Belgium).

## Results

### Overview of the entire study population

The demographic and clinical features of the participants included in the analysis are reported in Table 2. The population included a higher proportion of women (72.00%) than men (28.00%). Most of the participants (89.28%) were of Han ethnicity. Hypertension was the most frequent diagnosis (28.24%), followed by cardiovascular disease (5.99%). Overall, the general health status of the studied population was good, as the prevalence of severe diseases, such as stroke (2.28%) and cancer (0.40%), was very low.

**Table 2:** General characteristics of the participants in this study.

	Centenarians	Near-centenarians	Total
*n* (male/female)	966 (175/791)	787 (316/ 471)	1,753 (491/1,262)
Age (years)	85.21 ± 4.33	102.79 ± 2.71	94.89 ± 9.43
Body mass index	20.71 ± 3.80	18.13 ± 4.01	19.42 ± 4.11
Waist-hip ratio	0.89 ± 0.22	0.90 ± 0.13	0.89 ± 0.17
Ethnic Han (%)	89.54%	88.96%	89.28%
Systolic blood pressure (mmHg)	147.27 ± 23.97	152.88 ± 24.93	150.32 ± 24.65
Diastolic blood pressure (mmHg)	78.92 ± 12.83	75.65 ± 13.37	77.14 ± 13.23
Hemoglobin (g/L)	126.83 ± 17.99	113.11 ± 16.76	119.25 ± 18.61
Erythrocyte (mmc)	4.43 ± 0.64	4.01 ± 0.61	4.20 ± 0.66
Mean corpuscular volume (fl)	91.75 ± 8.77	90.40 ± 8.95	91.00 ± 8.89
Total protein (g/dL)	72.36 ± 4.99	68.67 ± 6.26	70.33 ± 6.01
Albumin (g/dL)	42.10 ± 3.35	38.44 ± 4.06	40.08 ± 4.17
Serum homocysteine (µmol/L)	24.66 ± 11.85	26.02 ± 13.48	25.38 ± 12.76
Serum uric acid (µmol/L)	340.50 ± 92.87	329.40 ± 100.38	334.38 ± 97.21
Serum urea (mmol/L)	5.70 ± 2.22	6.50 ± 3.30	6.14 ± 2.89
Serum creatinine (mg/dL)	0.96 ± 0.29	0.98 ± 0.41	0.97 ± 0.36
Disease	40.27%	39.14%	39.76%
Hypertension (%)	28.78%	27.58%	28.24%
Cardiovascular disease (%)	6.21%	5.72%	5.99%
Stroke (%)	2.80%	1.65%	2.28%
Diabetes (%)	2.38%	1.40%	1.94%
Dyslipidemia (%)	0.93%	0.88%	0.91%
Cancer (%)	0.41%	0.38%	0.4%

**Notes.**

Abbreviations MDRD modification of diet in renal disease CKD-EPI chronic kidney disease epidemiology collaboration BIS1 Berlin Initiative Study 1 eGFR estimated glomerular filtration rate.

### The incidence of various stages of CKD calculated using different equations

**Figure 1:**
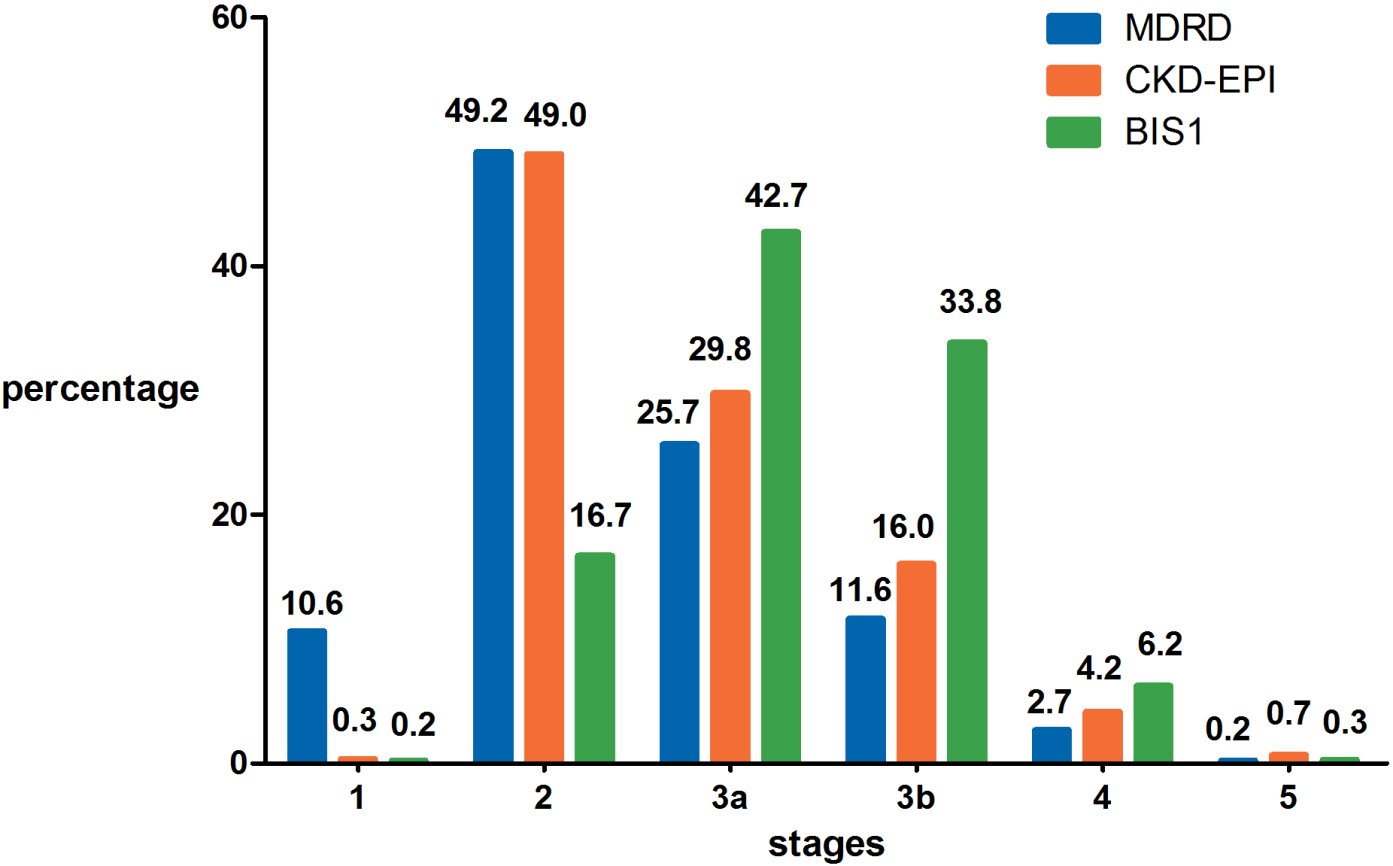
The distribution of CKD stages according to eGFR calculated by the MDRD, CKD-EPI and BIS1 equations. MDRD, modification of diet in renal disease; CKD-EPI, chronic kidney disease epidemiology collaboration; BIS1, Berlin Initiative Study 1.

The distributions of CKD stages according to the eGFR calculated by the MDRD, CKD-EPI and BIS1 equations are shown in Fig. 1. The highest incidence rate was observed for stages 2 and 3, while the other three stages had a relatively rare incidence. The results of CKD staging calculated using the MDRD and CKD-EPI equations were very similar. However, fewer patients were diagnosed with CKD stage 2 (16.7% vs 49.2% and 49.0%), and more patients were diagnosed with CKD stages 3a (42.7% vs 25.7% and 29.8%) and 3b (33.8% vs 11.6% and 16.0%) using the BIS1 equation than using the MDRD and CKD-EPI equations, respectively. More patients were diagnosed with CKD stage 1 using the MDRD equation than using the CKD-EPI and BIS1 equations (10.6% vs 0.3% and 0.2%, respectively).

### Important factors contributing to the differences in CKD staging based on different equations

Partial correlation coefficients and coefficients of partial determination are shown in Table 3. The Δ(MDRD, CKD-EPI), Δ(MDRD, BIS1) and Δ(CKD-EPI, BIS1) values were all significantly correlated with Scr (all *P* < 0.001). Δ(MDRD, CKD-EPI) and Δ(MDRD, BIS1) values were significantly correlated with age (all *P* < 0.001), but Δ(CKD-EPI, BIS1) values were not (*P* > 0.05). Scr levels had the greatest contribution to variability in Δ(MDRD, BIS1) and Δ(CKD-EPI, BIS1) values (partial *R*^2^, 55.354% and 53.436%, respectively). Age contributed less to variability in Δ(MDRD, CKD-EPI) and Δ(MDRD, BIS1) values (partial *R*^2^, 14.138% and 17.556%, respectively).

**Table 3:** Partial correlations of difference between GFR estimates obtained with MDRD, CKD-EPI, BIS1 equations and equation related factors (age and serum creatinine).

	Δ(MDRD, CKD-EPI)		Δ(MDRD, BIS1)		Δ(CKD-EPI, BIS1)	
	Partial correlation coefficient	Partial *R*2	Partial correlation coefficient	Partial *R*2	Partial correlation coefficient	Partial *R*2
Age (years)	0.376^*^	14.138%	0.419^*^	17.556%	0.025	0.063%
Serum creatinine (mg/dL)	−0.406^*^	16.484%	−0.744^*^	55.354%	−0.731^*^	53.436%

**Notes.**

* Significantly different from controls (*P* < 0.001).

Abbreviations MDRD modification of diet in renal disease CKD-EPI chronic kidney disease epidemiology collaboration BIS1 Berlin Initiative Study 1

### Detailed description of the effects of various factors on the results obtained from different equations

**Figure 2:**
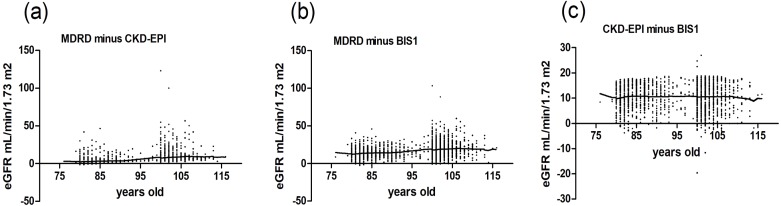
Scatter plot reflecting Δ(MDRD, CKD-EPI), Δ(MDRD, BIS1) and Δ(CKD-EPI, BIS1) according to age. MDRD, modification of diet in renal disease; CKD-EPI, chronic kidney disease epidemiology collaboration; BIS1, Berlin Initiative Study 1; Δ, difference. (A) Scatter plot reflecting Δ(MDRD, CKD-EPI) according to age; (B) Scatter plot reflecting Δ(MDRD, BIS1) according to age; (C) Scatter plot reflecting Δ(CKD-EPI, BIS1) according to age.

**Figure 3:**
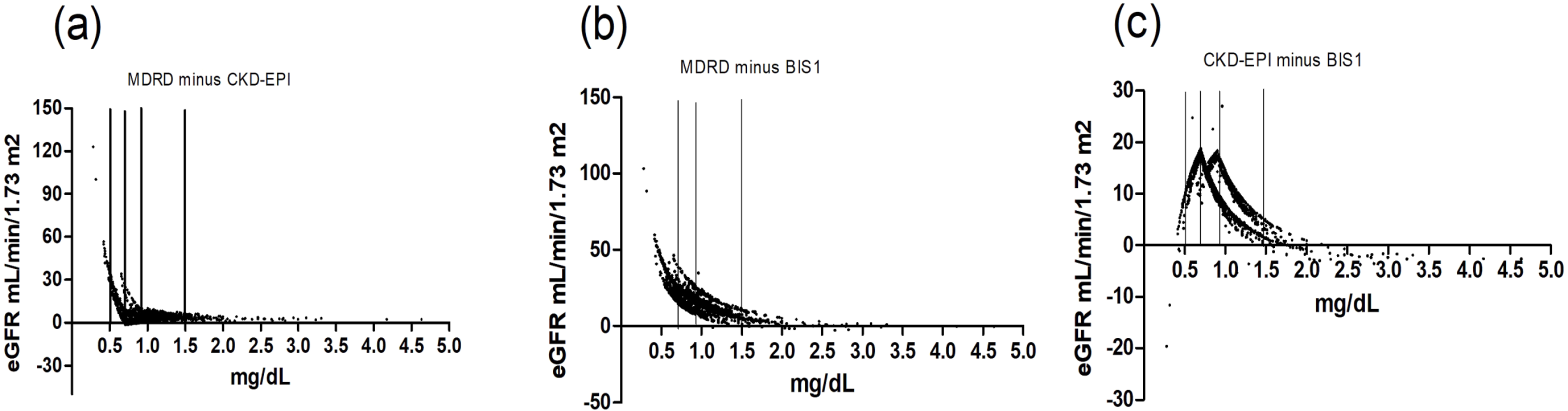
Scatter plot reflecting Δ(MDRD, CKD-EPI), Δ(MDRD, BIS1) and Δ(CKD-EPI, BIS1) according to Scr. The three vertical lines represent creatinine values of 0.7 mg/dL, 0.9 mg/dL and 1.5 mg/dL, respectively. MDRD, modification of diet in renal disease; CKD-EPI, chronic kidney disease epidemiology collaboration; BIS1, Berlin Initiative Study 1; Δ, difference. (A) Scatter plot reflecting Δ(MDRD, CKD-EPI) according to Scr; (B) Scatter plot reflecting Δ(MDRD, BIS1) according to Scr; (C) Scatter plot reflecting Δ(CKD-EPI, BIS1) according to Scr.

**Figure 4:**
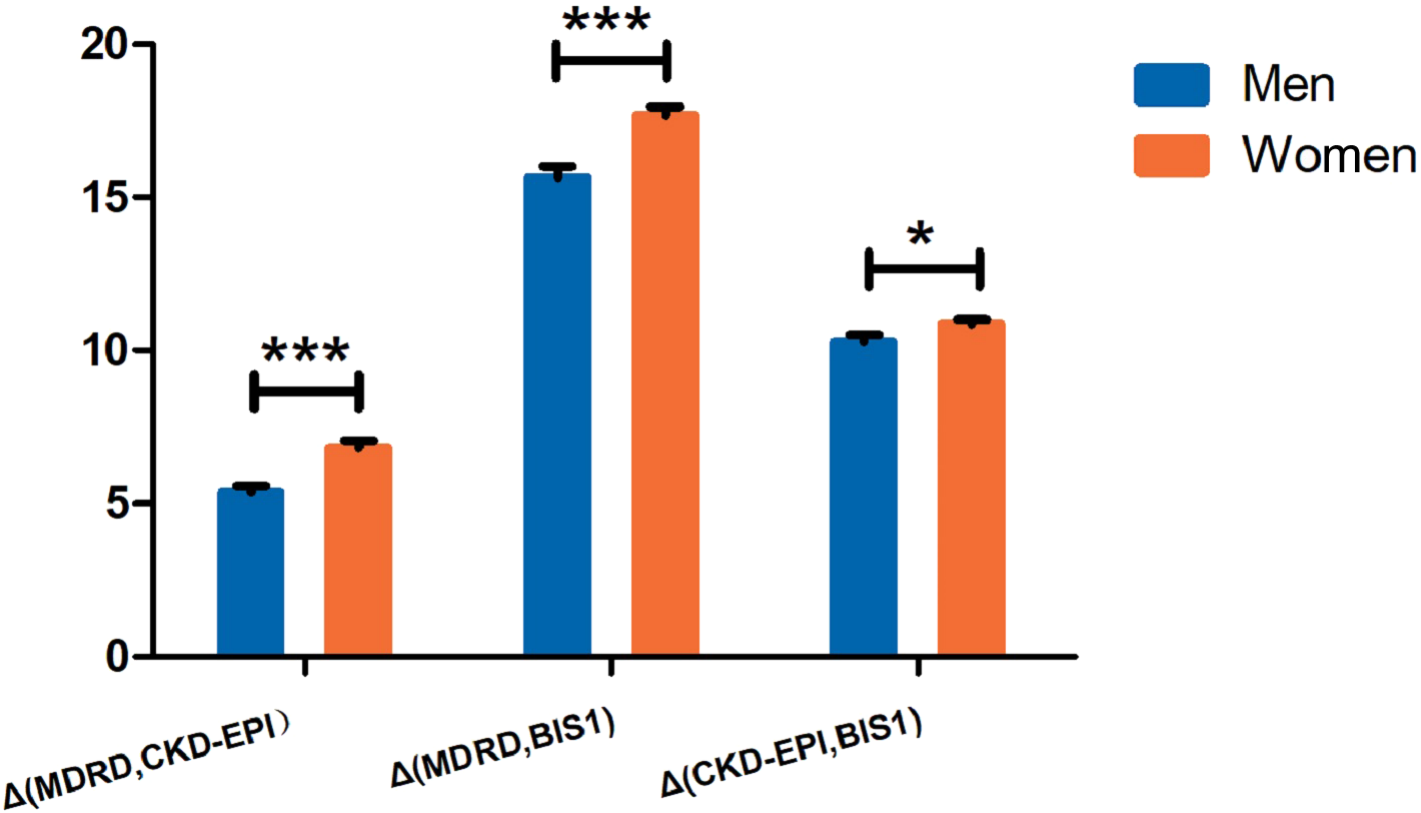
The differences between GFR estimates obtained with MDRD, CKD-EPI, BIS1 equations stratified by sex. *Significantly different from controls (*P* < 0.05), ***Significantly different from controls (*P* < 0.001). MDRD, modification of diet in renal disease; CKD-EPI, chronic kidney disease epidemiology collaboration; BIS1, Berlin Initiative Study 1.

Because age and Scr levels exerted the most important effects on the variability in these three equations, we drew a scatter plot reflecting Δ(MDRD, CKD-EPI), Δ(MDRD, BIS1) and Δ(CKD-EPI, BIS1) in groups stratified by age (Fig. 2) and Scr (Fig. 3). Differences existed across age groups. In addition, Δ(MDRD, CKD-EPI) and Δ(MDRD, BIS1) increased with age, but Δ(CKD-EPI, BIS1) was relatively stable with age. In addition, Δ(CKD-EPI, BIS1) exhibited the greatest difference (Fig. 2). As shown in Fig. 3, Δ(MDRD, CKD-EPI) and Δ(MDRD, BIS1) were considerable in subjects with Scr levels less than 0.7 mg/dL and decreased with the Scr level. A considerable difference between CKD-EPI and BIS1 was observed for participants with Scr levels ranging from 0.5 to 1.5 mg/dL. This difference increased with Scr levels ranging from 0.5 to 0.7 mg/dL, was relatively stable for Scr levels ranging from 0.7 to 0.9 mg/dL, and decreased with Scr levels ranging from 0.9 to 1.5 mg/dL. For groups stratified by sex, unpaired Student’s *t*-tests were used to investigate the effect of sex on differences between these three equations (Fig. 4). The differences in the three comparisons were all greater in female participants than in male participants (*p* < 0.05). In addition, Δ(MDRD, BIS1) exhibited the greatest difference in both males and females.

### Agreement of the three equations in groups stratified by age, Scr levels and sex

The results of the scatter plot were divided into four segments according to age (Table S1) and Scr levels (Table S2). In addition, we investigated the agreement of the three equations in groups stratified by sex (Table S3). We performed the CKD classification and *κ* analysis with different eGFR equations. The agreement in every age group was substantial (*κ* = 0.773, 0.761, 0.656 and 0.642) between the MDRD and CKD-EPI equations but fair (*κ* = 0.405, 0.287, 0.261 and 0.222) between the MDRD and BIS1 equations, and this agreement decreased with age. However, the agreement in every age group was moderate (*κ* = 0.527, 0.421, 0.459 and 0.394) between the CKD-EPI and BIS1 equations and was relatively stable with age.

In groups stratified by Scr levels, the agreement between the three comparisons was slight (*κ* = 0.035, 0.014 and 0.063) for Scr levels less than 0.7 mg/dL and substantial (*κ* = 0.640, 0.619 and 0.786) for Scr levels greater than 1.5 mg/dL; this agreement increased with the Scr levels.

In groups stratified by sex, the agreement among these three equations was very similar in both male and female participants. The agreement of the MDRD and CKD-EPI equations, the MDRD and BIS1 equations, and the CKD-EPI and BIS1 equations was substantial (*κ* = 0.706 and 0.700), moderate (*κ* = 0.458 and 0.501), and fair (*κ* = 0.248 and 0.319) in both male and female participants, respectively.

## Discussion

Along with the gradual increase in the aging population, the prevalence of CKD in the elderly population has increased annually (Zeng et al., 2017). The classification of different CKD stages in patients implies different clinical decisions (Inker et al., 2014). Thus, GFR estimation is important for detecting and staging CKD, determining drug dosages, stratifying risk and tailoring the dosages of several drugs to kidney function (Farrington et al., 2017). From the perspective of clinical practice, our aim was to estimate the agreement between the MDRD, CKD-EPI and BIS1 equations in terms of CKD staging to aid in assessing the impact of discrepancies in GFR calculated using these equations and seek the most appropriate equation for some specific groups of centenarians and near-centenarians.

The general health status of our studied population was good (Table 2), and the present study did not display subject selection bias. Our findings confirm that the BIS1 equation classified fewer patients with CKD stage 2 and more patients with CKD stages 3 and 4 than the MDRD and CKD-EPI equations (Fig. 1); specific diagnostic and therapeutic measures are recommended for patients with these CKD stages (Inker et al., 2014). Several important factors affect differences between GFR estimates obtained with the MDRD, CKD-EPI and BIS1 equations (Table 3). We observed a strong negative correlation between Scr levels and all three comparisons, particularly Δ(MDRD, BIS1) and Δ(CKD-EPI, BIS1), which explained 55.354% and 53.436% of the variability in these two comparisons, respectively. Age contributed less to variability in Δ(MDRD, CKD-EPI) and Δ(MDRD, BIS1) values (partial *R*^2^, 14.138% and 17.556%, respectively). A potential explanation for this finding is that many elderly people are diagnosed with sarcopenia and multiple comorbidities that cause a loss of muscle mass and thus low Scr levels (Tap et al., 2018; Tufan et al., 2017). On the other hand, these three equations were not developed using a large number of healthy people, resulting in a worse performance in elderly people with low Scr levels (Levey et al., 2006; Levey et al., 2009; Schaeffner et al., 2012).

More patients with CKD stage 1 and healthy people were identified using the MDRD equation than with the CKD-EPI and BIS1 equations, potentially resulting in an optimistic eGFR estimate for some elderly individuals (Fig. 1). Our scatter plots show differences across age groups (Fig. 2). These differences between the MDRD and CKD-EPI equations and between the MDRD and BIS1 equations increased with age, and the trend is more obvious, especially in those older than 95 years of age. Therefore, the eGFR tended to be more easily overestimated by the MDRD equation than by the CKD-EPI and BIS1 equations in elderly people, particularly centenarians. According to current diagnostic criteria, the renal function of a very small number of elderly people is considered a normal GFR (Stevens et al., 2010). However, the outcomes of older people with an eGFR less than 60 mL/min/1.73 m^2^ are quite different from those of younger patients (Esposito et al., 2012). These elderly individuals, particularly centenarians, usually show a slower CKD progression, potentially due to a physiological decrease in renal function that is not the result of kidney damage (John et al., 2004). Additionally, the incidence of adverse symptoms, such as anemia, hyperuricemia and hypertension, is lower in older people with an eGFR greater than 30 mL/min/1.73 m^2^ (John et al., 2004). Therefore, the GFR pathological threshold might be lower among older patients than among younger patients (Lattanzio et al., 2015). Among the three equations, the CKD staging results calculated with the MDRD equation are the most optimistic, especially for those older than 95 years of age (Table S1). In addition, in this particular elderly population, we do not need to be very strict with their GFR control. Therefore, we tend to suggest the use of the MDRD equation to calculate the GFR of elderly individuals >95 years old who have no risk factors for cardiovascular disease, considering physical and economic burdens.

Treatment during the earlier stages of CKD is effective at slowing the progression to kidney failure. The initiation of treatment for cardiovascular risk factors at earlier stages of CKD should be effective at reducing cardiovascular disease events both before and after the onset of kidney failure. Unfortunately, CKD is usually underdiagnosed and undertreated worldwide, resulting in lost opportunities for prevention. Treatment of the primary disease, regular follow-up, early diagnosis and treatment can reduce the mortality of CKD in the elderly. Among the three equations, the CKD staging results calculated with the BIS1 equation are the most pessimistic in elderly individuals aged younger than 94 years old (Table S1). Therefore, we tend to suggest the use of the BIS1 equation to calculate the GFR of elderly individuals aged younger than 94 years old who have risk factors for cardiovascular disease, considering the greater number of opportunities to treat and improve the long-term survival rates of these individuals.

On the other hand, the CKD-EPI equation classified more patients with CKD stage 5 than the other two equations, potentially resulting in a pessimistic eGFR estimate for them. According to the criteria advocated by the Kidney Disease Outcomes Quality Initiative (KDOQI), dialysis should be initiated in elderly patients with a GFR less than 20 mL/min/1.73 m^2^ combined with symptoms, water load and complications (National Kidney, 2002). Joly et al. (2003) reported a higher 1-year survival rate for elderly patients who chose dialysis treatment, and the survival time was extended by 20 months compared with patients with ESRD who did not receive dialysis. Among the three equations, the CKD staging results calculated with the CKD-EPI equation are the most pessimistic in the elderly participants (Table S2). Therefore, we tend to suggest the use of the CKD-EPI equation to calculate the GFR of elderly individuals with Scr levels greater than 1.5 mg/dL, with the aim of improving long-term survival rates.

In groups stratified by Scr levels (Fig. 3), considerable differences between the MDRD and CKD-EPI equations and between the MDRD and BIS1 equations were observed for participants with Scr levels less than 0.7 mg/dL, and these differences decreased with the Scr level. Therefore, the eGFR tended to be more easily overestimated by the MDRD equation than by the CKD-EPI and BIS1 equations for people with low Scr levels, particularly in participants with Scr levels less than 0.7 mg/dL. However, this is less important than at higher Scr levels because fewer important decisions are made when the eGFR is high. A considerable difference in the CKD-EPI and BIS1 equations was observed for participants with Scr levels ranging from 0.5 to 1.5 mg/dL. This difference increased in participants with Scr levels ranging from 0.5 to 0.7 mg/dL, was relatively stable for participants with Scr levels ranging from 0.7 to 0.9 mg/dL, and decreased for participants with Scr levels ranging from 0.9 to 1.5 mg/dL. Therefore, the eGFR tended to be more easily overestimated by the CKD-EPI equation than by the BIS1 equation for people with Scr levels ranging from 0.5 to 1.5 mg/dL.

In groups stratified by sex (Fig. 4), differences in these three equations were higher in female participants than in male participants, consistent with the findings of a previous study (Corsonello et al., 2017). Notably, the eGFR tended to be overestimated when these three equations were used in women. Older women often present with occult renal insufficiency, a separation of normal Scr levels and a decrease in the GFR (Rahimi et al., 2008). These clinical manifestations may be due to reduced levels of endogenous creatinine produced by the muscle and a lower than expected Scr level, which easily results in an overestimation of renal function (Keith et al., 2004). However, the GFR of older women decreased sharply in a process that accelerated with age and appeared to be nonlinear. According to Malmgren et al. (2015), older women with CKD exhibit a greater than 3-fold increased risk of death. Among the three equations, the CKD staging results calculated with the BIS1 equation are the most pessimistic in the elderly (Tables S2 and S3). Therefore, we tend to suggest the use of the BIS1 equation to calculate the GFR of older women with Scr levels less than 0.7 mg/dL, with the aim of avoiding an overestimation of kidney function due to reduced muscle mass.

The present study has some strengths. Undoubtedly, our sample of centenarians is very large, and this information is very important for clinicians worldwide. Indeed, the centralized and standardized serum analyses utilized in this study guarantee the quality of the data. Furthermore, we collected variables that facilitated the identification of sources of divergence among the equations.

The limitations of the present study also deserve consideration. First, the GFR cannot be measured directly because some centenarians were weak and were unable to undergo the complex gold standard diagnostic procedure. Therefore, it is difficult to use large-scale population studies to build a new equation for centenarians and near-centenarians. Second, we provided suggestions for specific types of centenarians and near-centenarians in particular situations by analyzing the differences among equations. However, this is indeed a complexity when patients cross a threshold and more in-depth research is needed to find further clinical evidence in the future. Third, the study individuals were all Chinese, so the results may not be generalizable to patients with other ethnic backgrounds or local lifestyles.

## Conclusions

In conclusion, these three equations should not be considered interchangeable for assessing eGFR in elderly individuals, particularly centenarians. The eGFR diverged significantly in the range of GFR values corresponding to CKD stages 2 and 3, which might dramatically impact clinical decision-making practices. Age, Scr levels and sex were the most important contributors to the variability in these three equations. The Scr level, age and sex of the patient should be combined when considering which equation to use to avoid overestimating or underestimating the GFR. The choice of the following equation may be more appropriate: use the MDRD equation to calculate the GFR of elderly individuals aged >95 years old who have no risk factors for cardiovascular disease, considering physical and economic burdens; use the BIS1 equation to calculate the GFR of elderly individuals aged less than 94 years old who have risk factors for cardiovascular disease, considering the greater number of opportunities to treat and improve the long-term survival rates of these patients; use the CKD-EPI equation to calculate the GFR of elderly individuals with Scr levels >1.5 mg/dL, with the aim of improving long-term survival rates; and use the BIS1 equation to calculate the GFR of older women with Scr levels less than 0.7 mg/dL, with the aim of avoiding an overestimation of kidney function due to reduced muscle mass.

##  Supplemental Information

10.7717/peerj.8636/supp-1 Data S1 Raw Data

10.7717/peerj.8636/supp-2 Table S1 Agreement of the three equations stratified by age Note: Shaded cells indicate patients with consistent CKD classifications across different equations. Abbreviations: MDRD, modification of diet in renal disease; CKD-EPI, chronic kidney disease epidemiology collaboration; BIS1, Berlin Initiative Study 1.

10.7717/peerj.8636/supp-3 Table S2 Agreement of the three equations stratified by serum creatinine Note: Shaded cells indicate patients with consistent CKD classifications across different equations. Abbreviations: MDRD, modification of diet in renal disease; CKD-EPI, chronic kidney disease epidemiology collaboration; BIS1, Berlin Initiative Study 1.

10.7717/peerj.8636/supp-4 Table S3 Agreement of the three equations stratified by sex Note: Shaded cells indicate patients with consistent CKD classifications across different equations. Abbreviations: MDRD, modification of diet in renal disease; CKD-EPI, chronic kidney disease epidemiology collaboration; BIS1, Berlin Initiative Study 1.

## Additional Information and Declarations

### Data Availability

The following information was supplied regarding data availability: The dataset is available in the Supplemental Files.
