# Supplementary material for: The practicality of different eGFR equations in centenarians and near-centenarians: which equation should we choose?
Source: PeerJ. 2020 Feb 21;8:e8636. doi: 10.7717/peerj.8636 (PMC7039118; doi:10.7717/peerj.8636)
Supplement: Table S1 — Note: Shaded cells indicate patients with consistent CKD classifications across different equations. Abbreviations: MDRD, modification of diet in renal disease; CKD-EPI, chronic kidney disease epidemiology collaboration; BIS1, Berlin Initiative Study 1. [file peerj-08-8636-s002.docx]

Table S1. Agreement of the three equations stratified by age.

| 79-84 years  (n=421) | | | | **MDRD** | | | | | | κ | P |
| --- | --- | --- | --- | --- | --- | --- | --- | --- | --- | --- | --- |
|  |  |  |  | Stage 1 | Stage 2 | Stage 3a | Stage 3b | Stage 4 | Stage 5 |  |  |
| **CKD-EPI** | | | Stage 1 | 3(5.8) | 0 | 0 | 0 | 0 | 0 | 0.773 | 0.022 |
|  |  |  | Stage 2 | 49(94.2) | 231(93.5) | 0 | 0 | 0 | 0 |  |  |
|  |  |  | Stage 3a | 0 | 16(6.5) | 87(94.6) | 0 | 0 | 0 |  |  |
|  |  |  | Stage 3b | 0 | 0 | 5(5.4) | 23(100.0) | 0 | 0 |  |  |
|  |  |  | Stage 4 | 0 | 0 | 0 | 0 | 7(100.0) | 0 |  |  |
|  |  |  | Stage 5 | 0 | 0 | 0 | 0 | 0 | 0 |  |  |
|  | | | | **MDRD** | | | | | | κ | P |
|  |  |  |  | Stage 1 | Stage 2 | Stage 3a | Stage 3b | Stage 4 | Stage 5 |  |  |
| **BIS1** | | | Stage 1 | 1(1.9) | 0 | 0 | 0 | 0 | 0 | 0.405 | 0.028 |
|  |  |  | Stage 2 | 51(98.1) | 99(40.1) | 0 | 0 | 0 | 0 |  |  |
|  |  |  | Stage 3a | 0 | 148(59.9) | 64(69.6) | 0 | 0 | 0 |  |  |
|  |  |  | Stage 3b | 0 | 0 | 28(30.4) | 23(100.0) | 0 | 0 |  |  |
|  |  |  | Stage 4 | 0 | 0 | 0 | 0 | 7(100.0) | 0 |  |  |
|  |  |  | Stage 5 | 0 | 0 | 0 | 0 | 0 | 0 |  |  |
|  | | | | **CKD-EPI** | | | | | | κ | P |
|  |  |  |  | Stage 1 | Stage 2 | Stage 3a | Stage 3b | Stage 4 | Stage 5 |  |  |
| **BIS1** | | Stage 1 | | 1(33.3) | 0 | 0 | 0 | 0 | 0 | 0.527 | 0.032 |
|  |  | Stage 2 | | 2(66.7) | 148(52.9) | 0 | 0 | 0 | 0 |  |  |
|  |  | Stage 3a | | 0 | 132(47.1) | 80(77.7) | 0 | 0 | 0 |  |  |
|  |  | Stage 3b | | 0 | 0 | 23(22.3) | 28(100.0) | 0 | 0 |  |  |
|  |  | Stage 4 | | 0 | 0 | 0 | 0 | 7(100.0) | 0 |  |  |
|  |  | Stage 5 | | 0 | 0 | 0 | 0 | 0 | 0 |  |  |
| 85-94 years  (n=336) | | | | **MDRD** | | | | | | κ | P |
|  |  |  |  | Stage 1 | Stage 2 | Stage 3a | Stage 3b | Stage 4 | Stage 5 |  |  |
| **CKD-EPI** | | Stage 1 | | 1(3.7) | 0 | 0 | 0 | 0 | 0 | 0.761 | 0.027 |
|  |  | Stage 2 | | 26(96.3) | 170(89.5) | 0 | 0 | 0 | 0 |  |  |
|  |  | Stage 3a | | 0 | 20(10.5) | 76(87.4) | 0 | 0 | 0 |  |  |
|  |  | Stage 3b | | 0 | 0 | 11(12.6) | 25(83.3) | 0 | 0 |  |  |
|  |  | Stage 4 | | 0 | 0 | 0 | 5(16.7) | 1(50.0) | 0 |  |  |
|  |  | Stage 5 | | 0 | 0 | 0 | 0 | 1(50.0) | 0 |  |  |
|  | | | | **MDRD** | | | | | | κ | P |
|  |  |  |  | Stage 1 | Stage 2 | Stage 3a | Stage 3b | Stage 4 | Stage 5 |  |  |
| **BIS1** | | Stage 1 | | 1(3.7) | 0 | 0 | 0 | 0 | 0 | 0.287 | 0.026 |
|  |  | Stage 2 | | 26(96.3) | 35(18.4) | 0 | 0 | 0 | 0 |  |  |
|  |  | Stage 3a | | 0 | 155(81.6) | 32(36.8) | 0 | 0 | 0 |  |  |
|  |  | Stage 3b | | 0 | 0 | 55(63.2) | 25(83.3) | 0 | 0 |  |  |
|  |  | Stage 4 | | 0 | 0 | 0 | 5(16.7) | 2(100.0) | 0 |  |  |
|  |  | Stage 5 | | 0 | 0 | 0 | 0 | 0 | 0 |  |  |
|  | | | | **CKD-EPI** | | | | | | κ | P |
|  |  |  |  | Stage 1 | Stage 2 | Stage 3a | Stage 3b | Stage 4 | Stage 5 |  |  |
| **BIS1** | | | Stage 1 | 1(100.0) | 0 | 0 | 0 | 0 | 0 | 0.421 | 0.031 |
|  |  |  | Stage 2 | 0 | 61(31.1) | 0 | 0 | 0 | 0 |  |  |
|  |  |  | Stage 3a | 0 | 135(68.9) | 52(54.2) | 0 | 0 | 0 |  |  |
|  |  |  | Stage 3b | 0 | 0 | 44(45.8) | 36(100.0) | 0 | 0 |  |  |
|  |  |  | Stage 4 | 0 | 0 | 0 | 0 | 6(100.0) | 1(100.0) |  |  |
|  |  |  | Stage 5 | 0 | 0 | 0 | 0 | 0 | 0 |  |  |
| 95-104 years  (n=795) | | | | **MDRD** | | | | | | κ | P |
|  |  |  |  | Stage 1 | Stage 2 | Stage 3a | Stage 3b | Stage 4 | Stage 5 |  |  |
| **CKD-EPI** | | | Stage 1 | 2(2.3) | 0 | 0 | 0 | 0 | 0 | 0.656 | 0.020 |
|  |  |  | Stage 2 | 86(97.7) | 228(67.1) | 0 | 0 | 0 | 0 |  |  |
|  |  |  | Stage 3a | 0 | 112(32.9) | 142(65.1) | 0 | 0 | 0 |  |  |
|  |  |  | Stage 3b | 0 | 0 | 76(34.9) | 93(79.5) | 0 | 0 |  |  |
|  |  |  | Stage 4 | 0 | 0 | 0 | 24(20.5) | 24(85.7) | 0 |  |  |
|  |  |  | Stage 5 | 0 | 0 | 0 | 0 | 4(14.3) | 4(100.0) |  |  |
|  | | | | **MDRD** | | | | | | κ | P |
|  |  |  |  | Stage 1 | Stage 2 | Stage 3a | Stage 3b | Stage 4 | Stage 5 |  |  |
| **BIS1** | | | Stage 1 | 2(2.3) | 0 | 0 | 0 | 0 | 0 | 0.261 | 0.017 |
|  |  |  | Stage 2 | 67(76.1) | 1(0.3) | 0 | 0 | 0 | 0 |  |  |
|  |  |  | Stage 3a | 19(21.6) | 272(80.0) | 2(0.9) | 0 | 0 | 0 |  |  |
|  |  |  | Stage 3b | 0 | 67(19.7) | 216(99.1) | 79(67.5) | 0 | 0 |  |  |
|  |  |  | Stage 4 | 0 | 0 | 0 | 38(32.5) | 28(100.0) | 0 |  |  |
|  |  |  | Stage 5 | 0 | 0 | 0 | 0 | 0 | 4(100.0) |  |  |
|  | | | | **CKD-EPI** | | | | | | κ | P |
|  |  |  |  | Stage 1 | Stage 2 | Stage 3a | Stage 3b | Stage 4 | Stage 5 |  |  |
| **BIS1** | | Stage 1 | | 2(100.0) | 0 | 0 | 0 | 0 | 0 | 0.459 | 0.021 |
|  |  | Stage 2 | | 0 | 68(21.7) | 0 | 0 | 0 | 0 |  |  |
|  |  | Stage 3a | | 0 | 245(78.0) | 47(18.5) | 1(0.6) | 0 | 0 |  |  |
|  |  | Stage 3b | | 0 | 1(0.3) | 207(81.5) | 154(91.1) | 0 | 0 |  |  |
|  |  | Stage 4 | | 0 | 0 | 0 | 14(8.3) | 48(100.0) | 4(50.0) |  |  |
|  |  | Stage 5 | | 0 | 0 | 0 | 0 | 0 | 4(50.0) |  |  |
| 105-116 years  (n=201) | | | | **MDRD** | | | | | | κ | P |
|  |  |  |  | Stage 1 | Stage 2 | Stage 3a | Stage 3b | Stage 4 | Stage 5 |  |  |
| **CKD-EPI** | | Stage 1 | | 0 | 0 | 0 | 0 | 0 | 0 | 0.642 | 0.041 |
|  |  | Stage 2 | | 19(100.0) | 50(58.8) | 0 | 0 | 0 | 0 |  |  |
|  |  | Stage 3a | | 0 | 35(41.2) | 34(64.2) | 0 | 0 | 0 |  |  |
|  |  | Stage 3b | | 0 | 0 | 19(35.8) | 29(85.3) | 0 | 0 |  |  |
|  |  | Stage 4 | | 0 | 0 | 0 | 5(14.7) | 7(70.0) | 0 |  |  |
|  |  | Stage 5 | | 0 | 0 | 0 | 0 | 3(30.0) | 0 |  |  |
|  | | | | **MDRD** | | | | | | κ | P |
|  |  |  |  | Stage 1 | Stage 2 | Stage 3a | Stage 3b | Stage 4 | Stage 5 |  |  |
| **BIS1** | Stage 1 | | | 0 | 0 | 0 | 0 | 0 | 0 | 0.222 | 0.038 |
|  | Stage 2 | | | 14(73.7) | 0 | 0 | 0 | 0 | 0 |  |  |
|  | Stage 3a | | | 5(26.3) | 52(61.2) | 0 | 0 | 0 | 0 |  |  |
|  | Stage 3b | | | 0 | 33(38.8) | 53(100.0) | 14(41.2) | 0 | 0 |  |  |
|  | Stage 4 | | | 0 | 0 | 0 | 20(58.8) | 9(90.0) | 0 |  |  |
|  | Stage 5 | | | 0 | 0 | 0 | 0 | 1(10.0) | 0 |  |  |
|  | | | | **CKD-EPI** | | | | | | κ | P |
|  |  |  |  | Stage 1 | Stage 2 | Stage 3a | Stage 3b | Stage 4 | Stage 5 |  |  |
| **BIS1** | | Stage 1 | | 0 | 0 | 0 | 0 | 0 | 0 | 0.394 | 0.029 |
|  |  | Stage 2 | | 0 | 14(20.3) | 0 | 0 | 0 | 0 |  |  |
|  |  | Stage 3a | | 0 | 55(79.7) | 2(2.9) | 0 | 0 | 0 |  |  |
|  |  | Stage 3b | | 0 | 0 | 67(97.1) | 33(68.8) | 0 | 0 |  |  |
|  |  | Stage 4 | | 0 | 0 | 0 | 15(31.2) | 12(100.0) | 2(66.7) |  |  |
|  |  | Stage 5 | | 0 | 0 | 0 | 0 | 0 | 1(33.3) |  |  |

Note: Shaded cells indicate patients with consistent CKD classifications across different equations. Abbreviations: MDRD, modification of diet in renal disease; CKD-EPI, chronic kidney disease epidemiology collaboration; BIS1, Berlin Initiative Study 1.
