# Supplementary material for: The practicality of different eGFR equations in centenarians and near-centenarians: which equation should we choose?
Source: PeerJ. 2020 Feb 21;8:e8636. doi: 10.7717/peerj.8636 (PMC7039118; doi:10.7717/peerj.8636)
Supplement: Table S2 — Note: Shaded cells indicate patients with consistent CKD classifications across different equations. Abbreviations: MDRD, modification of diet in renal disease; CKD-EPI, chronic kidney disease epidemiology collaboration; BIS1, Berlin Initiative Study 1. [file peerj-08-8636-s003.docx]

Table S2. Agreement of the three equations stratified by serum creatinine.

| Scr<0.7 mg/dl  (n=313) | | **MDRD** | | | | | | k | P |
| --- | --- | --- | --- | --- | --- | --- | --- | --- | --- |
|  |  | Stage 1 | Stage 2 | Stage 3a | Stage 3b | Stage 4 | Stage 5 |  |  |
| **CKD-EPI** | Stage 1 | 6(100) | 158(51.5) | 0 | 0 | 0 | 0 | 0.035 | 0.039 |
|  | Stage 2 | 0 | 149(48.5) | 0 | 0 | 0 | 0 |  |  |
|  | Stage 3a | 0 | 0 | 0 | 0 | 0 | 0 |  |  |
|  | Stage 3b | 0 | 0 | 0 | 0 | 0 | 0 |  |  |
|  | Stage 4 | 0 | 0 | 0 | 0 | 0 | 0 |  |  |
|  | Stage 5 | 0 | 0 | 0 | 0 | 0 | 0 |  |  |
|  | | **MDRD** | | | | | | k | P |
|  |  | Stage 1 | Stage 2 | Stage 3a | Stage 3b | Stage 4 | Stage 5 |  |  |
| **BIS1** | Stage 1 | 4(2.4) | 0 | 0 | 0 | 0 | 0 | 0.014 | 0.048 |
|  | Stage 2 | 140(85.4) | 55(36.9) | 0 | 0 | 0 | 0 |  |  |
|  | Stage 3a | 20(12.2) | 94(63.1) | 0 | 0 | 0 | 0 |  |  |
|  | Stage 3b | 0 | 0 | 0 | 0 | 0 | 0 |  |  |
|  | Stage 4 | 0 | 0 | 0 | 0 | 0 | 0 |  |  |
|  | Stage 5 | 0 | 0 | 0 | 0 | 0 | 0 |  |  |
|  | | **CKD-EPI** | | | | | | k | P |
|  |  | Stage 1 | Stage 2 | Stage 3a | Stage 3b | Stage 4 | Stage 5 |  |  |
| **BIS1** | Stage 1 | 4(66.7) | 0 | 0 | 0 | 0 | 0 | 0.063 | 0.043 |
|  | Stage 2 | 2(33.3) | 193(62.9) | 0 | 0 | 0 | 0 |  |  |
|  | Stage 3a | 0 | 114(37.1) | 0 | 0 | 0 | 0 |  |  |
|  | Stage 3b | 0 | 0 | 0 | 0 | 0 | 0 |  |  |
|  | Stage 4 | 0 | 0 | 0 | 0 | 0 | 0 |  |  |
|  | Stage 5 | 0 | 0 | 0 | 0 | 0 | 0 |  |  |
| 0.7≤Scr<0.9mg/dl  (n=792) | | **MDRD** | | | | | | k | P |
|  |  | Stage 1 | Stage 2 | Stage 3a | Stage 3b | Stage 4 | Stage 5 |  |  |
| **CKD-EPI** | Stage 1 | 0 | 0 | 0 | 0 | 0 | 0 | 0.597 | 0.018 |
|  | Stage 2 | 0 | 22(4.6) | 126(40.9) | 0 | 0 | 0 |  |  |
|  | Stage 3a | 0 | 458(95.4) | 182(59.1) | 4(100.0) | 0 | 0 |  |  |
|  | Stage 3b | 0 | 0 | 0 | 0 | 0 | 0 |  |  |
|  | Stage 4 | 0 | 0 | 0 | 0 | 0 | 0 |  |  |
|  | Stage 5 | 0 | 0 | 0 | 0 | 0 | 0 |  |  |
|  | | **MDRD** | | | | | | k | P |
|  |  | Stage 1 | Stage 2 | Stage 3a | Stage 3b | Stage 4 | Stage 5 |  |  |
| **BIS1** | Stage 1 | 0 | 0 | 0 | 0 | 0 | 0 | 0.058 | 0.027 |
|  | Stage 2 | 18(81.8) | 80(13.7) | 0 | 0 | 0 | 0 |  |  |
|  | Stage 3a | 4(18.2) | 431(73.8) | 44(23.7) | 0 | 0 | 0 |  |  |
|  | Stage 3b | 0 | 73(12.5) | 142(76.3) | 0 | 0 | 0 |  |  |
|  | Stage 4 | 0 | 0 | 0 | 0 | 0 | 0 |  |  |
|  | Stage 5 | 0 | 0 | 0 | 0 | 0 | 0 |  |  |
|  | | **CKD-EPI** | | | | | | k | P |
|  |  | Stage 1 | Stage 2 | Stage 3a | Stage 3b | Stage 4 | Stage 5 |  |  |
| **BIS1** | Stage 1 | 0 | 0 | 0 | 0 | 0 | 0 | 0.123 | 0.032 |
|  | Stage 2 | 0 | 98(20.4) | 0 | 0 | 0 | 0 |  |  |
|  | Stage 3a | 0 | 381(79.4) | 98(31.8) | 0 | 0 | 0 |  |  |
|  | Stage 3b | 0 | 1(0.2) | 210(68.2) | 4(100) | 0 | 0 |  |  |
|  | Stage 4 | 0 | 0 | 0 | 0 | 0 | 0 |  |  |
|  | Stage 5 | 0 | 0 | 0 | 0 | 0 | 0 |  |  |
| 0.9≤Scr<1.5mg/dl  (n=545) | | **MDRD** | | | | | | k | P |
|  |  | Stage 1 | Stage 2 | Stage 3a | Stage 3b | Stage 4 | Stage 5 |  |  |
| **CKD-EPI** | Stage 1 | 0 | 0 | 0 | 0 | 0 | 0 | 0.597 | 0.018 |
|  | Stage 2 | 0 | 72(100.0) | 57(26.6) | 0 | 0 | 0 |  |  |
|  | Stage 3a | 0 | 0 | 157(73.4) | 107(44.4) | 18(100.0) | 0 |  |  |
|  | Stage 3b | 0 | 0 | 0 | 134(55.6) | 0 | 0 |  |  |
|  | Stage 4 | 0 | 0 | 0 | 0 | 0 | 0 |  |  |
|  | Stage 5 | 0 | 0 | 0 | 0 | 0 | 0 |  |  |
|  | | **MDRD** | | | | | | k | P |
|  |  | Stage 1 | Stage 2 | Stage 3a | Stage 3b | Stage 4 | Stage 5 |  |  |
| **BIS1** | Stage 1 | 0 | 0 | 0 | 0 | 0 | 0 | 0.176 | 0.034 |
|  | Stage 2 | 0 | 102(79.1) | 54(20.5) | 0 | 0 | 0 |  |  |
|  | Stage 3a | 0 | 27(20.9) | 210(79.5) | 0 | 0 | 0 |  |  |
|  | Stage 3b | 0 | 0 | 0 | 111(73.0) | 0 | 0 |  |  |
|  | Stage 4 | 0 | 0 | 0 | 41(27.0) | 0 | 0 |  |  |
|  | Stage 5 | 0 | 0 | 0 | 0 | 0 | 0 |  |  |
|  | | **CKD-EPI** | | | | | | k | P |
|  |  | Stage 1 | Stage 2 | Stage 3a | Stage 3b | Stage 4 | Stage 5 |  |  |
| **BIS1** | Stage 1 | 0 | 0 | 0 | 0 | 0 | 0 | 0.442 | 0.021 |
|  | Stage 2 | 0 | 0 | 0 | 0 | 0 | 0 |  |  |
|  | Stage 3a | 0 | 72(100.0) | 83(38.8) | 1(0.4) | 0 | 0 |  |  |
|  | Stage 3b | 0 | 0 | 131(61.2) | 217(90.0) | 0 | 0 |  |  |
|  | Stage 4 | 0 | 0 | 0 | 23(9.5) | 18(100.0) | 0 |  |  |
|  | Stage 5 | 0 | 0 | 0 | 0 | 0 | 0 |  |  |
| Scr≥1.5mg/dl  (n=103) | | **MDRD** | | | | | | k | P |
|  |  | Stage 1 | Stage 2 | Stage 3a | Stage 3b | Stage 4 | Stage 5 |  |  |
| **CKD-EPI** | Stage 1 | 0 | 0 | 0 | 0 | 0 | 0 | 0.640 | 0.016 |
|  | Stage 2 | 0 | 0 | 0 | 0 | 0 | 0 |  |  |
|  | Stage 3a | 0 | 0 | 0 | 0 | 0 | 0 |  |  |
|  | Stage 3b | 0 | 0 | 0 | 36(69.2) | 0 | 0 |  |  |
|  | Stage 4 | 0 | 0 | 0 | 16(30.8) | 39(83.0) | 0 |  |  |
|  | Stage 5 | 0 | 0 | 0 | 0 | 8(17.0) | 4(100.0) |  |  |
|  | | **MDRD** | | | | | | k | P |
|  |  | Stage 1 | Stage 2 | Stage 3a | Stage 3b | Stage 4 | Stage 5 |  |  |
| **BIS1** | Stage 1 | 0 | 0 | 0 | 0 | 0 | 0 | 0.619 | 0.006 |
|  | Stage 2 | 0 | 0 | 0 | 0 | 0 | 0 |  |  |
|  | Stage 3a | 0 | 0 | 0 | 0 | 0 | 0 |  |  |
|  | Stage 3b | 0 | 0 | 0 | 30(57.7) | 0 | 0 |  |  |
|  | Stage 4 | 0 | 0 | 0 | 22(42.3) | 46(97.9) | 0 |  |  |
|  | Stage 5 | 0 | 0 | 0 | 0 | 1(2.1) | 4(100.0) |  |  |
|  | | **CKD-EPI** | | | | | | k | P |
|  |  | Stage 1 | Stage 2 | Stage 3a | Stage 3b | Stage 4 | Stage 5 |  |  |
| **BIS1** | Stage 1 | 0 | 0 | 0 | 0 | 0 | 0 | 0.786 | 0.003 |
|  | Stage 2 | 0 | 0 | 0 | 0 | 0 | 0 |  |  |
|  | Stage 3a | 0 | 0 | 0 | 0 | 0 | 0 |  |  |
|  | Stage 3b | 0 | 0 | 0 | 30(83.3) | 0 | 0 |  |  |
|  | Stage 4 | 0 | 0 | 0 | 6(16.7) | 55(100.0) | 7(58.3) |  |  |
|  | Stage 5 | 0 | 0 | 0 | 0 | 0 | 5(41.7) |  |  |

Note: Shaded cells indicate patients with consistent CKD classifications across different equations. Abbreviations: MDRD, modification of diet in renal disease; CKD-EPI, chronic kidney disease epidemiology collaboration; BIS1, Berlin Initiative Study 1. Abbreviations: serum creatinine, Scr.
