# Supplementary material for: The practicality of different eGFR equations in centenarians and near-centenarians: which equation should we choose?
Source: PeerJ. 2020 Feb 21;8:e8636. doi: 10.7717/peerj.8636 (PMC7039118; doi:10.7717/peerj.8636)
Supplement: Table S3 — Note: Shaded cells indicate patients with consistent CKD classifications across different equations. Abbreviations: MDRD, modification of diet in renal disease; CKD-EPI, chronic kidney disease epidemiology collaboration; BIS1, Berlin Initiative Study 1. [file peerj-08-8636-s004.docx]

Table S3. Agreement of the three equations stratified by sex

| Men  (n=491) | | | | **MDRD** | | | | | | k | P |
| --- | --- | --- | --- | --- | --- | --- | --- | --- | --- | --- | --- |
|  |  |  |  | Stage 1 | Stage 2 | Stage 3a | Stage 3b | Stage 4 | Stage 5 |  |  |
| **CKD-EPI** | | Stage 1 | | 0 | 31(13.5) | 0 | 0 | 0 | 0 | 0.706 | 0.008 |
|  |  | Stage 2 | | 0 | 199(86.5) | 58(34.9) | 0 | 0 | 0 |  |  |
|  |  | Stage 3a | | 0 | 0 | 108(65.1) | 33(40.7) | 0 | 0 |  |  |
|  |  | Stage 3b | | 0 | 0 | 0 | 48 (59.3) | 6(54.5) | 0 |  |  |
|  |  | Stage 4 | | 0 | 0 | 0 | 0 | 5(45.5) | 1(33.3) |  |  |
|  |  | Stage 5 | | 0 | 0 | 0 | 0 | 0 | 2(66.7) |  |  |
|  | | | | **MDRD** | | | | | | k | P |
|  |  |  |  | Stage 1 | Stage 2 | Stage 3a | Stage 3b | Stage 4 | Stage 5 |  |  |
| **BIS1** | | Stage 1 | | 0 | 0 | 0 | 0 | 0 | 0 | 0.458 | 0.021 |
|  |  | Stage 2 | | 0 | 69(30.0) | 0 | 0 | 0 | 0 |  |  |
|  |  | Stage 3a | | 0 | 160(69.6) | 75(45.2) | 1(1.2) | 0 | 0 |  |  |
|  |  | Stage 3b | | 0 | 1(0.4) | 91(54.8) | 74(91.4) | 0 | 0 |  |  |
|  |  | Stage 4 | | 0 | 0 | 0 | 6(7.4) | 11(100) | 1(33.3) |  |  |
|  |  | Stage 5 | | 0 | 0 | 0 | 0 | 0 | 2(66.7) |  |  |
|  | | | | **CKD-EPI** | | | | | | k | P |
|  |  |  |  | Stage 1 | Stage 2 | Stage 3a | Stage 3b | Stage 4 | Stage 5 |  |  |
| **BIS1** | | Stage 1 | | 0 | 0 | 0 | 0 | 0 | 0 | 0.284 | 0.029 |
|  |  | Stage 2 | | 27(87.1) | 42(16.3) | 0 | 0 | 0 | 0 |  |  |
|  |  | Stage 3a | | 4(12.9) | 187(72.8) | 45(31.9) | 0 | 0 | 0 |  |  |
|  |  | Stage 3b | | 0 | 28(10.9) | 96(68.1) | 42(77.8) | 0 | 0 |  |  |
|  |  | Stage 4 | | 0 | 0 | 0 | 12(22.2) | 6(100) | 0 |  |  |
|  |  | Stage 5 | | 0 | 0 | 0 | 0 | 0 | 2(100) |  |  |
| Women  (n=1262) | | | | **MDRD** | | | | | | k | P |
|  |  |  |  | Stage 1 | Stage 2 | Stage 3a | Stage 3b | Stage 4 | Stage 5 |  |  |
| **CKD-EPI** | | Stage 1 | | 6(100) | 149(23.7) | 0 | 0 | 0 | 0 | 0.700 | 0.012 |
|  |  | Stage 2 | | 0 | 480(76.3) | 125(35.1) | 0 | 0 | 0 |  |  |
|  |  | Stage 3a | | 0 | 0 | 231(64.9) | 78(39) | 0 | 0 |  |  |
|  |  | Stage 3b | | 0 | 0 | 0 | 122(61) | 28(45.2) | 0 |  |  |
|  |  | Stage 4 | | 0 | 0 | 0 | 0 | 34(54.8) | 7(77.8) |  |  |
|  |  | Stage 5 | | 0 | 0 | 0 | 0 | 0 | 2(22.2) |  |  |
|  | | | | **MDRD** | | | | | | k | P |
|  |  |  |  | Stage 1 | Stage 2 | Stage 3a | Stage 3b | Stage 4 | Stage 5 |  |  |
| **BIS1** | Stage 1 | | | 4(66.7) | 0 | 0 | 0 | 0 | 0 | 0.501 | 0.019 |
|  | Stage 2 | | | 2(33.3) | 222(35.3) | 0 | 0 | 0 | 0 |  |  |
|  | Stage 3a | | | 0 | 407(64.7) | 106(29.8) | 0 | 0 | 0 |  |  |
|  | Stage 3b | | | 0 | 0 | 250(70.2) | 177(88.5) | 0 | 0 |  |  |
|  | Stage 4 | | | 0 | 0 | 0 | 23(11.5) | 62(100) | 6(66.7) |  |  |
|  | Stage 5 | | | 0 | 0 | 0 | 0 | 0 | 3(33.3) |  |  |
|  | | | | **CKD-EPI** | | | | | | k | P |
|  |  |  |  | Stage 1 | Stage 2 | Stage 3a | Stage 3b | Stage 4 | Stage 5 |  |  |
| **BIS1** | | | Stage 1 | 4(2.6) | 0 | 0 | 0 | 0 | 0 | 0.319 | 0.022 |
|  |  |  | Stage 2 | 131(84.5) | 93(15.4) | 0 | 0 | 0 | 0 |  |  |
|  |  |  | Stage 3a | 20(12.9) | 440(72.7) | 53(17.2) | 0 | 0 | 0 |  |  |
|  |  |  | Stage 3b | 0 | 72(11.9) | 256(82.8) | 99(66.0) | 0 | 0 |  |  |
|  |  |  | Stage 4 | 0 | 0 | 0 | 51(34.0) | 40(97.6) | 0 |  |  |
|  |  |  | Stage 5 | 0 | 0 | 0 | 0 | 1(2.4) | 2(100) |  |  |

Note: Shaded cells indicate patients with consistent CKD classifications across different equations. Abbreviations: MDRD, modification of diet in renal disease; CKD-EPI, chronic kidney disease epidemiology collaboration; BIS1, Berlin Initiative Study 1.
